# IoT-Based Smart Garbage System for Efficient Food Waste Management

**DOI:** 10.1155/2014/646953

**Published:** 2014-08-28

**Authors:** Insung Hong, Sunghoi Park, Beomseok Lee, Jaekeun Lee, Daebeom Jeong, Sehyun Park

**Affiliations:** School of Electrical and Electronics Engineering, Chung-Ang University, Seoul 151-756, Republic of Korea

## Abstract

Owing to a paradigm shift toward Internet of Things (IoT), researches into IoT services have been conducted in a wide range of fields. As a major application field of IoT, waste management has become one such issue. The absence of efficient waste management has caused serious environmental problems and cost issues. Therefore, in this paper, an IoT-based smart garbage system (SGS) is proposed to reduce the amount of food waste. In an SGS, battery-based smart garbage bins (SGBs) exchange information with each other using wireless mesh networks, and a router and server collect and analyze the information for service provisioning. Furthermore, the SGS includes various IoT techniques considering user convenience and increases the battery lifetime through two types of energy-efficient operations of the SGBs: stand-alone operation and cooperation-based operation. The proposed SGS had been operated as a pilot project in Gangnam district, Seoul, Republic of Korea, for a one-year period. The experiment showed that the average amount of food waste could be reduced by 33%.

## 1. Introduction

The Internet of Things (IoT) is a concept in which surrounding objects are connected through wired and wireless networks without user intervention. In the field of IoT, the objects communicate and exchange information to provide advanced intelligent services for users. Owing to the recent advances in mobile devices equipped with various sensors and communication modules, together with communication network technologies such as Wi-Fi and LTE, the IoT has gained considerable academic interests.

The term Internet of Things was introduced by Kevin Ashton, who was the director of the Auto-ID Center of MIT in 1999 [1]. The initial technical realization of IoT was achieved by utilizing RFID technology for the identification and tracking of devices and storing device information. However, IoT utilizing RFID technology was limited to object tracking and extracting information of specific objects. The current IoT performs sensing, actuating, data gathering, storing, and processing by connecting physical or virtual devices to the Internet. For IoT applications performing these functions, a variety of researches on IoT services including environmental monitoring [2, 3], object tracking [4], traffic management [5], health care [6], and smart home technology [7, 8] are being conducted.

Owing to the characteristics and merits of IoT services, waste management has also become a significant issue in academia, industry, and government as major IoT application fields. An indiscriminate and illegal discharge of waste, an absence of waste disposal and management systems, and inefficient waste management policies have caused serious environmental problems and have incurred considerable costs for waste disposal. To handle these problems, various researches into waste management based on IoT technology have been conducted, from studies on RFID technology to studies on waste management platforms and systems [9–13]. However, there remains a lack of research into waste management based on IoT technology or on the application of developed waste management systems in Republic of Korea.

This paper proposes an IoT-based smart garbage system (SGS) composed of a number of smart garbage bins (SGBs), routers, and servers. Each SGB, which plays a role in collecting food waste, is battery operated for mobility and, considering the convenience to residents, performs various techniques through wireless communication. The server collects and analyzes the status of all SGBs and resident information collected through RFID readers. The router is used for server load distribution. The proposed system had been operated as a pilot project in Gangnam district, which is one of the local districts in Seoul, the capital of Republic of Korea, according to the food waste reduction policy of the Korean government. Through the proposed system, not only food waste is reduced but also residents and the government save costs.

The rest of this paper is organized as follows.  Section 2 describes the motivation for creating the IoT-based SGS.  Section 3 details the architecture of the SGS and the discharge process.  Section 4 presents the main techniques of the SGS. Next, Section 5 shows the implementation of the SGS in Gangnam district for a one-year period and the operation results. Finally, some concluding remarks and directions for future work are given in Section 6.

## 2. Motivation and Background

In existing food waste management, local governments manage food waste by deploying food waste bins and employing multiple pickup businesses for food waste collection. However, the existing food waste management method is based on a flat rate, that is, a price structure that charges a single fixed fee, which causes environmental problems and increases waste discharge because there are no restrictions on heavy producers of food waste and no incentives for lighter producers. Because food waste producers do not have a direct burden of expense for generating waste, it is difficult for their waste amounts to be efficiently reduced. Moreover, the low reliability of statistics on food waste has caused difficulty in adjusting and managing discharge amounts because a local government hires multiple pickup businesses for waste collection, and each of them uses a different measuring method.

To deal with these problems in existing food waste management, a volume-rate garbage disposal system has been introduced. In particular, in Republic of Korea, three types of volume-rate garbage disposal systems, that is, chips and stickers, standard plastic garbage bags, and RFID-based garbage collection systems, are currently being used.  Table 1 describes the pros and cons of these three types of volume-rate garbage disposal systems. The most significant difference among them is that an accurate discharge weight can be obtained for an RFID-based garbage collection system when collecting food waste, which is difficult to measure accurately for chips and stickers and standard plastic garbage bags. For example, for standard plastic garbage bags, the weight of each bag may differ according to the resident's discharge habits and contents. In a chip and sticker method, although a collection box is used, thereby decreasing the allowable tolerance, accurate weight data also cannot be provided. Measuring accurate weight data is important, because of providing disposal convenience, after collecting and imposing the right duty for discharging how much food garbage they throw away.

In an RFID-based garbage collection system, an RFID collection bin includes a communication module for communicating with a central server, an RFID tag module for reading the data from an RFID card, automatic garbage entrance, and a scale function to measure the weight of the food waste. However, the collection bin communicates only with a server and lacks machine-to-machine communication with the other collection bins, which may cause a server overload. Furthermore, owing to the delay incurred from the complex discharge process of an RFID-based garbage collection system, users have a lengthy wait; in addition, an RFID-based garbage collection system lacks mobility because of a fixed power supply, causing further user inconvenience.

To solve these problems in existing RFID-based garbage collection systems, an IoT-based SGS is proposed. The proposed SGS fits into the category of IoT applied to external and public environments and was therefore designed to include the necessary components for such applications.

*(i) Reliability.* In IoT applied to external and public environments, communication is important for service provisioning. In particular, since this type of IoT has a wide service domain, reliable communication is necessary for devices to communicate with each other. Therefore, the SGBs utilized in the proposed system communicate with each other based on a wireless mesh network (WMN), securing communication reliability.

*(ii) Mobility.* IoT devices in an external environment may need to move on occasion. For a high level of mobility, the proposed system operates with a battery instead of the fixed power source that an existing RFID card system utilizes. With a battery-based power supply, the mobility of the proposed system is secured.

*(iii) Service Continuity.* In IoT with a wide service domain, data exchanges and services should be conducted seamlessly at any time and any location. Thus, SGBs, which communicate and exchange information based on a WMN, enable users to discharge their food waste anywhere a bin is available.

*(iv) User Convenience.* User convenience has been enhanced with the advent of IoT. For user convenience, the proposed SGS reduces the process delay time of the existing RFID-based garbage collection systems, which enables users to discharge their food waste without a lengthy wait.

*(v) Energy Efficiency.* IoT applied to external and public environments relies on an always-on infrastructure and requires mobility, causing a large amount of energy consumption. To solve this problem, the SGBs operate using energy-efficient techniques, increasing their battery lifetimes.

## 3. Smart Garbage System Architecture

### 3.1. Architecture Overview

**Figure 1:**
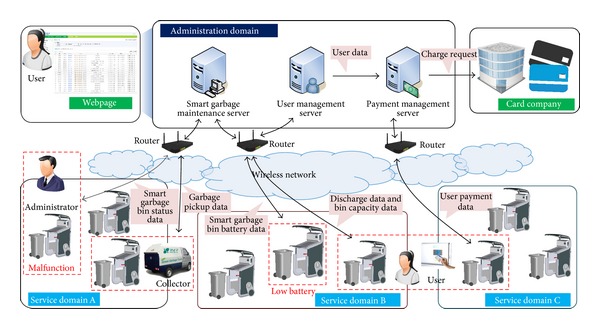
Overview of smart garbage system.

The architecture of the SGS is shown in Figure 1. The SGBs, which are installed near apartment buildings and individual houses, exchange information with each other and send the information to the server through wireless communication. Structurally, the proposed system is divided into two domains: an administration domain and a service domain. In the administration domain, information transferred from a SGB is analyzed and processed. In the service domain, residents throw away their food waste in a SGB, and resident and SGB information is collected and transferred to the administration domain.

*(i) Administration Domain.* In this domain, registered resident information, payment information, and status information, such as the battery life, memory, and any malfunctions of the SGBs, are collected. To achieve this, three servers are used: a smart garbage maintenance server, a user management server, and a payment management server. The user management server manages food waste discharge information and the personal information of the registered residents who are registered in the user management server through an administrator. Furthermore, information on the discharge amount of food waste is stored and classified based on region, resident, and bin in the user management server. The charge management server conducts the payment process based on the weight of the food waste with the resident's card company. When a resident uses an RFID card to discharge his food waste, his personal card information registered on the RFID card is transferred to the charge management server, which then requests the card company to process the payment. The smart garbage maintenance server plays a role in managing all information related to the SGBs such as the amount of food waste each SGB has, the amount of food waste a collection company has gathered, and the status information of the SGBs. Thus, if a malfunction is detected in a SGB after analyzing the status information, an administrator is sent to check the problem, and the smart garbage maintenance server induces residents to use a nearby SGB. All information managed in the administration domain is also provided through a Web-based service, through which the administrators can determine the state of the system and residents can check the amount of food waste they have thrown away and for how much they have paid.

**Figure 2:**
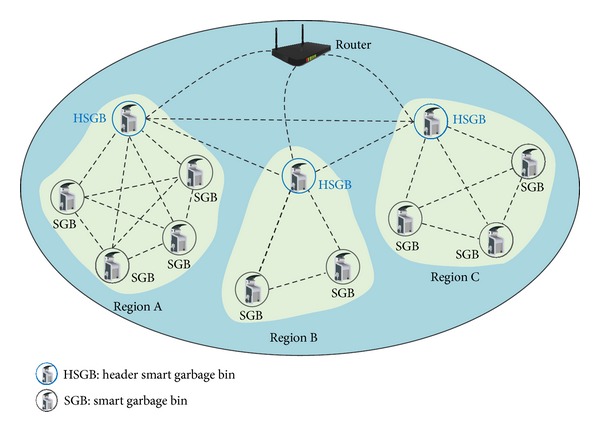
Network topology of smart garbage bins.

*(ii) Service Domain.* This domain is where the residents throw away their food waste. When a resident's RFID card touches the RFID reader of a SGB, the SGB authenticates the resident and opens the lid. The resident then throws away his food waste, and the SGB measures its weight. After the discharge process, the SGB sends the collected information on the resident and the weight of his food waste to the administration domain. Based on the collected information, a garbage collector collects the food waste from the SGB, an administrator inspects or repairs the bin, and a cleaner cleans the bin as necessary.  Figure 2 illustrates the network topology of SGBs located in the service domain. The SGBs exchange information such as their capacity, battery life, and resident information through a WMN. Therefore, service continuity is guaranteed even when the same residents use different garbage bins. A header smart garbage bin (HSGB), located within each region, analyzes and manages the other SGBs within its region after collecting their information. The HSGB also exchanges this information with other HSGBs through the WMN, allowing the service continuity to be secured. Furthermore, for network reliability, if a communication problem occurs in a HSGB, header authority is delegated to the most appropriate SGB within the same region.

### 3.2. Discharge Process of Smart Garbage System

**Figure 3:**
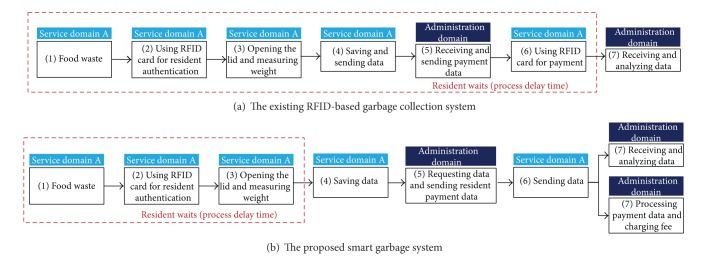
Discharge process of existing RFID-based garbage collection system and the proposed smart garbage system.

As mentioned above, the proposed system uses a new discharge process to minimize the delay caused by the payment and data transmission processes.  Figure 3 shows a comparison between an existing RFID-based garbage collection system and the proposed system. In the existing RFID-based garbage collection system, a resident touches his RFID card to the garbage bin twice. The first touch is for resident authentication, and the second touch is for his payment. Because a data transmission between a garbage bin and a server is required before payment, the process delay incurred from the moment the food waste is weighed until the fee is paid may be lengthy, and residents may be inconvenienced. In the proposed system, however, food waste disposal and the payment process are conducted by touching an RFID card to the SGB only once, thereby reducing the process delay of existing RFID-based garbage collection systems. After the resident authentication and weighing process, the balance of the RFID card is shown on an LCD screen of the SGB using the payment data previously received from the server and the present weight of the food waste. This marks the end of the discharge process requiring the residents to wait. Then, if no other residents are waiting to use the SGB, the SGB then sends the payment data to the server through a router each time it receives a request message from the router, and the server processes the payment data of all residents and charges their fees through their credit card company. Using this discharge process, an additional RFID card touch for payment is unnecessary, which reduces the process delay.

### 3.3. Middleware Architecture of Smart Garbage System

**Figure 4:**
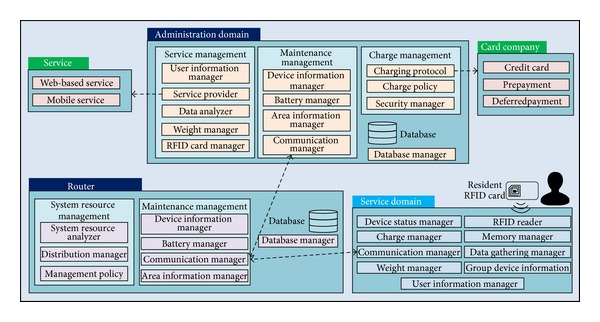
Middleware architecture of the proposed smart garbage system.

Figure 4 describes the entire middleware architecture of SGS. The service is based on the cooperation between the central server in the administration domain and a SGB in the service domain. The router shown in the figure is included in the administrator domain and acts as a distributed server for supplementing the centralized server's weakness when increasing the number of SGBs. Multiple routers are arranged to minimize the load and manage traffic through the server according to the number of SGBs in service.

The centralized server in the administration domain is composed of the three modules: service management, maintenance management, and charge management modules.

*(i) Service Management Module.* The service management module is based on the information obtained from a SGB, and it includes a user information manager for inputting or modifying the user information, a service provider to provide Web-based and mobile services, a data analyzer for analyzing information for compiling statistics, a weight manager for determining the unit price of the food waste, and an RFID card manager for managing the RFID card information.

*(ii) Maintenance Management Module.* This management module is composed of a device information manager to deal with information on each SGB, a battery manager to check the battery status of the SGBs, a communication manager to manage the communication status, and an area information manager for management of the area information.

*(iii) Charge Management Module.* This module deals with information regarding the charge process by the SGB and includes three components: a charge protocol to cooperate with an external charge interface, a charge policy component to determine the charge policy according to prepayment and deferred payment policies, and security management for encryption of the charge information.

In addition to these three modules, a database and database manager are used, the latter of which was designed for providing information required by the server or router.

Although the router normally performs maintenance management, it only takes allocated SGBs even though the server deals with all SGBs. A description of the system resource management can be given as follows.

*System Resource Management Module.* This module monitors the resource status of each SGB and the other routers and includes a distributed manager, which gives a specific role to each SGB based on analyzed information regarding the status of the battery and memory, and the management policy for system resource distribution. For example, if one SGB is unserviceable, the system resource management sends the necessary information to the SGB to guide residents to neighboring SGBs.

In addition to the system resource management and maintenance management, the database manager in the router cooperates with the database manager on the server and receives the required data on the allocated SGBs.

The middleware of a SGB, located in the bottom layer of the system architecture, is composed of a device status manager module to check the status of the SGB, a weight manager to measure the weight of the inserted food waste, and a data gathering manager to process the data received from other SGBs, the router, or the server.

## 4. IoT Techniques of Smart Garbage System

### 4.1. Energy-Efficient Stand-Alone Operation of a Smart Garbage Bin

Owing to the battery-based power supply for a SGB, both basic and low-power operations of a SGB are required to improve the battery efficiency. Existing RFID-based garbage collection systems powered from electric wires are consistently in always-on mode for users. Moreover, whenever a discharge process is conducted, the bins communicate with the server for a data update. However, in the case of a battery-based SGS, there is a problem of inefficient energy use if the proposed system is used in exactly the same manner as an existing electricity-based system. Therefore, the proposed system uses an energy-efficient communication technique for battery saving.

**Figure 5:**
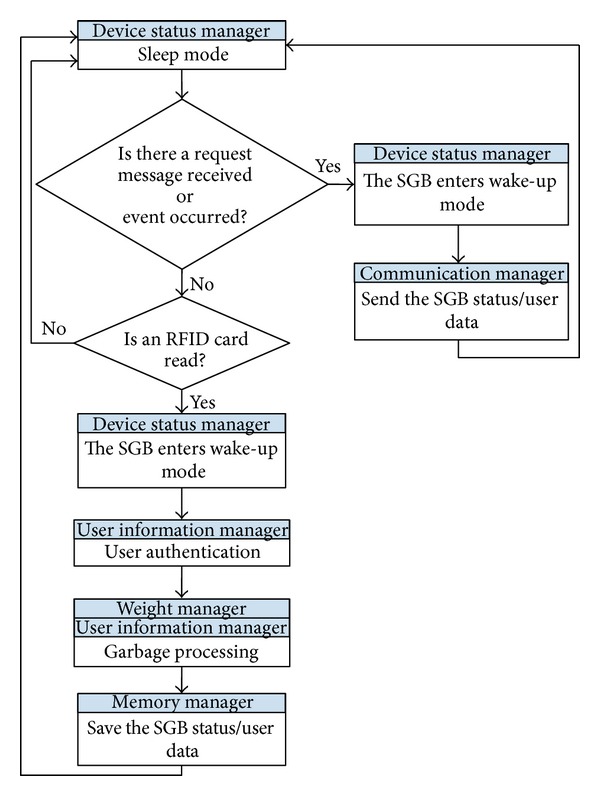
Flowchart of energy-efficient stand-alone operation of a smart garbage bin.

Figure 5 shows a flowchart of an energy-efficient stand-alone operation of a SGB.

*(i) Process 1.* The SGBs remain in sleep mode for low-power operation. However, because a SGB should be ready to receive a request message from a router or device data from SGBs in the same region, the communication module is always turned on.

*(ii) Process 2.* There are three different cases of this type of process.

 Case 1: a router sends a request message requiring the status information of the SGBs and resident information to the HSGB 12 times a day for a data update of the SGS. Thus, if the HSGB receives a request message from the router, the HSGB enters wake-up mode and sends all information on the residents and SGBs within the same region to the router.
 Case 2: if a SGB receives a request message from another SGB in the same region, the SGB enters wake-up mode and sends the requested information to the SGB that sent the message.
 Case 3: the HSGB can detect events such as communication problems and a lack of capacity or battery life. Therefore, if the HSGB detects events occurring in another SGB within its region, the HSGB enters wake-up mode and sends the event information to a router without a request message from the router.

In each of these cases, the SGB or the HSGB enters sleep mode for low-power operation. 

*(iii) Process 3.* In addition to the communication module, the RFID reader of a SGB is also always in an on state, allowing it to read a resident's RFID card at any time. Because the RFID system is event-driven, if the RFID reader of a SGB reads a resident's RFID card, the SGB enters wake-up mode and conducts user authentication and the garbage discharge process. Then, without sending any information, the SGB stores the information and reenters sleep mode.

### 4.2. Energy-Efficient Cooperation-Based Operation of a Smart Garbage Bin

In addition to their energy-efficient stand-alone operation, the SGBs operate in an energy-efficient manner by cooperating with each other. A router chooses a HSGB according to the battery and memory status of each SGB in the region, and the HSGB then collects information from the other SGBs. However, because the SGBs operate using a battery, a problem may occur if there is only one SGB operating as a header bin. To address this problem, the system resource management in the router checks the status of the SGBs and delegates the header authority to the SGB that has the largest amount of battery life and is the least used.

For example, take SGBs A, B, and C, with SGB A being the HSGB. SGB A is chosen to be the HSGB because it has more battery life than bins B and C and, as the least used bin, is expected to consume less energy. However, during operation, if the SGB A becomes frequently used and is thereby expected to consume a significant amount of energy, the router compares its expected power consumption and battery status with that of SGBs B and C. The router then delegates the header authority to either bin B or C accordingly.

To accomplish this process, the system resource management calculates the expected battery time of a SGB through (1). Consider

(1) $$\begin{array}{c} \frac{P_{B}}{\left(\sum_{i=1}^{7}P_{c}\left(i\right)+P_{w}\left(i\right)\right)/7}=d. \end{array}$$

**Figure 6:**
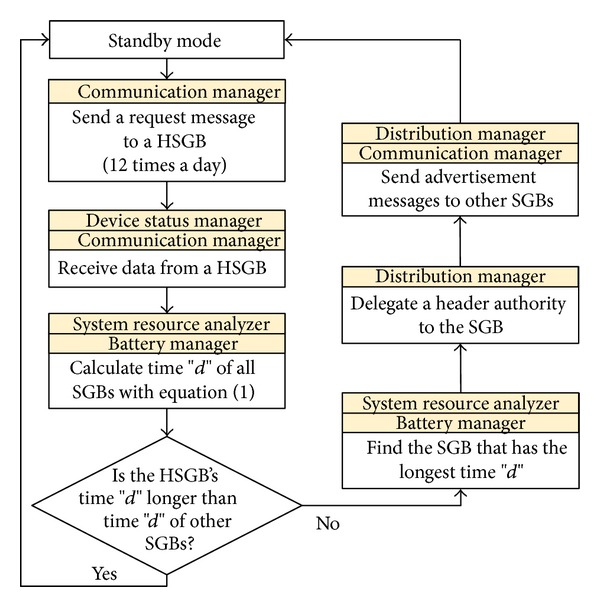
Flowchart of energy-efficient cooperation-based operation of a smart garbage bin.

*P*_*B*_ represents the current state of charge of a battery, *P*_*c*_(*i*) is the power consumption required for communication per day, and *P*_*w*_(*i*) is the power consumption for device operation per day. Based on the power consumption for seven days and the *P*_*B*_, the expected battery use time, *d*, of a SGB can be calculated. Therefore, based on time *d* calculated by the router, one of the SGBs, A, B, or C, becomes the HSGB.  Figure 6 shows a flowchart of an energy-efficient cooperation-based operation of a SGB.

### 4.3. Adaptive User-Oriented Charge Policy

The objective of the SGS is a reduction in food waste and efficient garbage management. Even if the residents are motivated to reduce their food waste after seeing the discharge process, expecting all residents to do so may be unrealistic because the cost reduction is low.

To motivate residents to reduce their food waste, the proposed SGS applies an adaptive user-oriented charge policy in place of charging fees per kg of food waste. The basic idea of the adaptive user-oriented charge policy is that the unit cost of food waste per kg is decreased if the food waste amount of a particular month is reduced compared to the amount of the previous month.

For example, take users A and B, where user A's food waste amount for the last month was 20 kg. Therefore, if user A is charged 20,000 Korean won, the unit cost for food waste per kg is 1,000 won. However, if A's food waste amount for the current month is 18 kg, which is a 10% reduction from last month, next month's unit cost for food waste per kg will be 900 won, which is also a 10% reduction from the basic unit cost. In the case of user B, his food waste amount for last month was also 20 kg. However, if his food waste amount for the current month is 22 kg, which is a 10% increase from the previous month, their next month's unit cost for food waste per kg will be 1,100 won, which is a 10% increase from the basic unit cost. The charge policy applied to the proposed SGS can be defined through the following equation:

(2) Base  Rate+(Waste  EmissionCWaste  EmissionP) ×Past  Changable  Rate=Next  Month  Rate

(3) $$\begin{array}{c} \text{Next}\;\text{Month}\;\text{Rate}-\text{Base}\;\text{Rate}=\text{Past}\;\text{Changable}\;\text{Rate}. \end{array}$$

Base Rate is the basic monthly charge, Waste  Emission_*C*_ is the food waste amount for this month, Waste  Emission_*P*_ is the food waste amount for last month, and Past Changeable Rate is the monthly changing charge. Based on this equation, next month's unit cost of food waste per kg is applied to the residents.

Furthermore, if the capacity of SGBs in a resident region is full, the SGBs show the available SGB list on their LCD screen. In this case, since the residents have to use a SGB in another region, an additional incentive, that is, a 10% reduction in unit cost, is given to the residents to compensate for their inconvenience.

### 4.4. Food Waste Collection Path and Number Optimization

In existing food waste management, multiple pickup businesses are employed to collect food waste, and these pickup businesses do so from midnight to early morning using several collection vehicles. However, since these vehicles move along a fixed route and the collectors are unable to know the amount of food waste that needs to be collected, unnecessary collections may occur.

**Figure 7:**
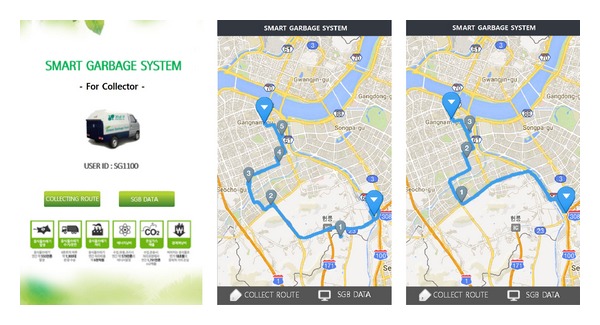
Mobile application for the collectors.

The proposed SGS proposes an efficient food waste collection system by monitoring the capacity of the SGBs. When the collectors request the status information of the SGBs along their collection route from the server through a smartphone, the server provides the information on the location and number of SGBs that need to be collected by utilizing the area information in the server middleware to the collector's smartphone.  Figure 7 shows a mobile application that uses an open-map API. The mobile application shows the location of the SGBs that need to be collected, as well as the optimized collection path generated based on the status information of the SGBs.

Food waste collection is commonly conducted once per day. However, in a commercial area where there is more food waste than in other locations, the food waste collection should be performed more than once per day. The server therefore adjusts the food waste collection time based on the total amount of food waste accumulated over the past seven days:

(4) $$\begin{array}{c} \overset{24}{\underset{t=1}{\sum }}\frac{E_{t}}{k}=S,\;\;\left(\overset{24}{\underset{t=1}{\sum }}\frac{E_{a}}{7}=E_{t}\right), \end{array}$$

where *E*_*t*_ is average discharge amount of food waste per hour, *k* is capacity of a smart garbage bin, *S* is number of food waste collection, and ∑*E*_*a*_ is total amount of food waste at a certain time.

The above equation is for the food waste collection time interval. Based on the average discharge amount of food waste per hour, the number of food waste collections is calculated. Using the value of *S*, the SGS adjusts the collection time and establishes efficient collection plans.

### 4.5. Event-Based IoT Techniques for the Smart Garbage System

**Figure 8:**
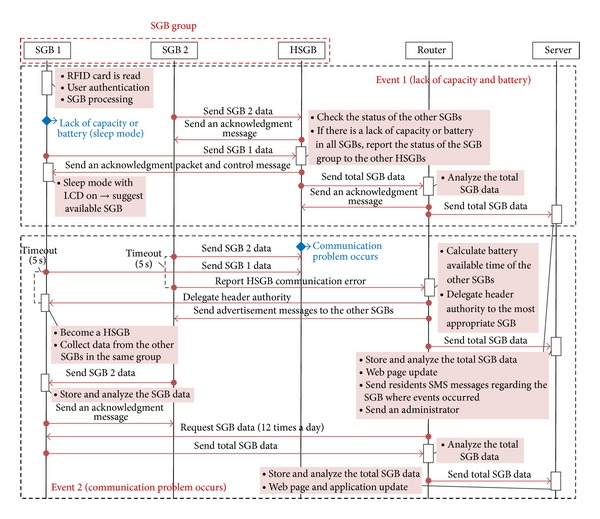
Sequence diagram for operation of the smart garbage system for two different events.

User convenience should be considered as the first priority in the operation of SGBs. Therefore, for service continuity and user convenience, the SGBs should induce the residents to use them by cooperating with each other when events such as a lack of capacity or battery occur. Furthermore, when a communication problem occurs in a HSGB, the header authority is delegated to another SGB for communication reliability. A sequence diagram for the operation of the SGS for two different events is illustrated in Figure 8.

*(i) Event 1: Lack of Capacity or Battery.* In an existing RFID-based garbage collection system, residents may be unable to discharge their food waste owing to a lack of capacity or when the garbage bins are turned off during the discharge process because of a lack of battery power. The proposed system, however, can prevent such events before they occur. After the discharge process, a SGB stores its status information. At this time, if the capacity of the SGB exceeds 90% or if the battery life drops below 5%, the SGB sends its status information to the HSGB and enters sleep mode. The HSGB, which has received the status information, then checks the other SGBs within the same region. The HSGB then sends a control message and the status information of the other SGBs to the SGB in which an event occurred to show a list of available SGBs on the LCD screen. In addition, the HSGB sends all information on its group of SGBs to the server through a router. The server then updates the Web page, sends SMS messages to the residents located in the area where the event has occurred, and sends an administrator to take action.

*(ii) Event 2: A Communication Problem Occurs.* If a communication problem occurs in a certain SGB, the problem can be detected when SGBs communicate with each other. The communication problem is then reported to the server through the HSGB. However, in the case of a communication problem in the HSGB, the header authority should be delegated to another SGB. Thus, if a SGB does not receive an acknowledgement message from the HSGB within 5 seconds after sending data, the SGB detects that a communication problem has occurred in the HSGB. The first SGB that detects the problem reports the situation to the router. The router then calculates the available battery life of the other SGBs in the same region and delegates the header authority to the most appropriate bin.

## 5. System Implementation and Experimental Results

### 5.1. Hardware Structure of a Smart Garbage Bin

Table 2 shows the specifications of a SGB. A load cell for measuring the weight of the food waste is located at the bottom of each SGB. The full size of a SGB was determined by considering the height of the users. Furthermore, for mobility, a lithium-ion battery is utilized as a power supply. However, depending on the circumstances, a SGB can use a fixed power source.

**Figure 9:**
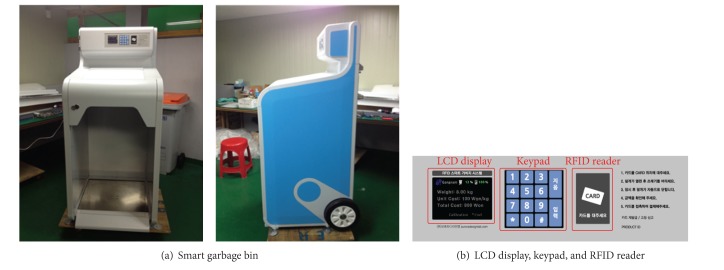
(a) Smart garbage bin and (b) LCD display, keypad, and RFID reader.

The hardware structure of a SGB is composed of eight modules: load cell, main system, interface, modem, motor, LCD display, AD converter, and RFID reader. First, the load cell [14] measures the initial analog value and sends it to the main system through the AD converter module attached to the main system. The AD converter module converts the analog value into a digital value. The value processed by the AD converter module is converted into a weight result in the main system. During this process, the characteristic of the load cell is not linear according to the weight change, and thus it should be corrected in the main system. The weight result is transferred to the interface. The interface sends the result to the modem or LCD display as demanded. Moreover, the interface also manages all the operations in the SGB, such as analyzing the input data from the RFID reader and driving the motor to open or close the lid of the bin. The actual SGB is shown in Figure 9.

### 5.2. System Implementation

**Figure 10:**
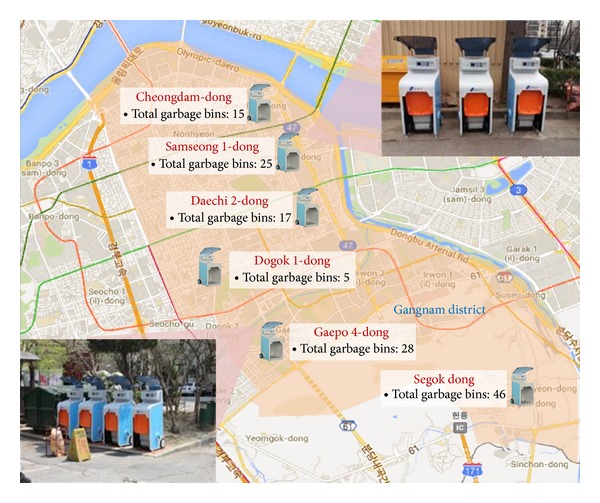
Implementation of a smart garbage system.

The proposed SGS had been operated as a pilot project in Gangnam district. In total, 136 SGBs were deployed in Gangnam's six subdistricts. The bins were applied to apartment housing areas in five of the subdistricts and to detached housing areas in the other district.  Figure 10 shows the locations where the SGBs were deployed, their number, and the system implementation.

As shown in Figure 10, a SGB is structured with a conventional food waste bin placed inside. System implementation was performed by simply placing the SGB at the location where a conventional food waste bin was previously located and fixing the conventional food waste bin inside the smart bin. In addition, since the SGB operates on battery power, additional construction connecting it to a neighboring commercial electricity line was unnecessary.

**Figure 11:**
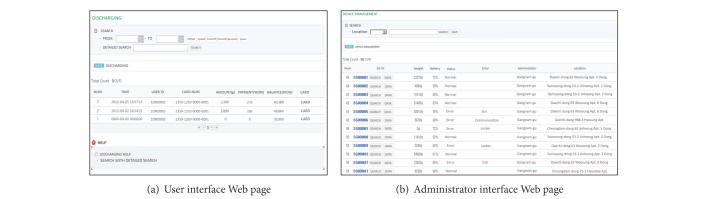
Web-based food waste management service.

The Web-based service structure is presented in Figure 11. The SGS provided an ID and password to each user for their RFID card and Web-based service. The users were divided into three classes: an administrator, collector, and the residents. The administrator can check the present and accumulated amount of food waste of each SGB, the status of all SGBs, and the time log. The administrator is then able to classify the information based on region, resident, and SGB. Moreover, a service enabling new users and RFID cards to be registered was provided to the administrator. While the administrator is given complete authority, a resident can only check the discharge amount of their food waste and payment information, and the collector can check the status of the collected food waste and receive a notification whenever the capacity of a SGB exceeds 90%.

### 5.3. Experimental Results

**Figure 12:**
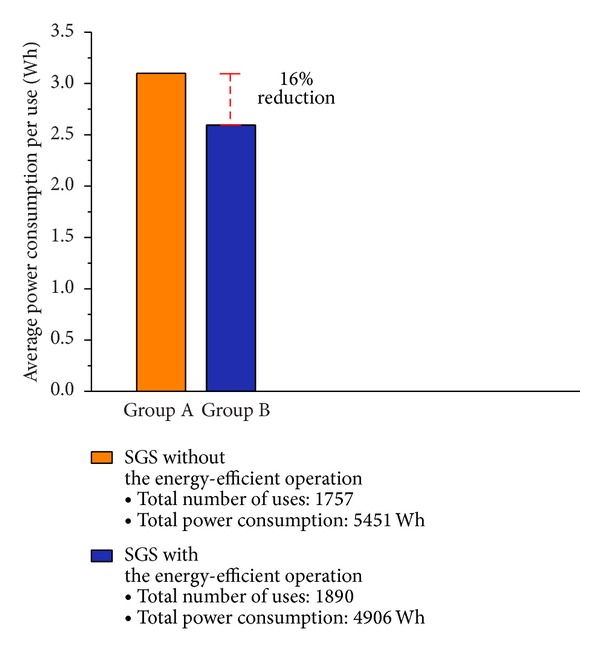
Comparison of average power consumption per use in Groups A and B.

*(i) Energy Efficiency*. To verify the energy efficiency of stand-alone and cooperation-based operations, two comparison groups, A and B, were set up. Group A had ten SGBs operating in the same manner as an existing RFID-based garbage collection system. Therefore, group A was normally kept in sleep mode, and it entered wake-up mode whenever a user utilized a bin. Group A conducted the discharge process and communicated with the server every time this process was finished. Furthermore, the SGBs in group A communicated with the router, and no header bin was used. For group B, which also had ten SGBs, a HSGB was delegated by the router and was changed to another bin depending on the battery status of all SGBs in the area. The two groups were used for a two-week period. To generate identical experimental conditions, two locations with a similar number of users and households were selected. The experimental results of Groups A and B are detailed in Tables 3 and 4, respectively. And Figure 12 shows the comparison of average power consumption per use in Groups A and B.

From the results, when the same number of service provisions and the same quality are assumed, the energy efficiency improved by 16% for the SGB group that applied an energy-efficient operation. Although there was little effect on the energy efficiency of device operation when including the opening of the lid and the use of an LCD screen, energy efficiency improvement was achieved by controlling the amount of communication with the server or router and applying an energy-efficient operation.

*(ii) Food Waste Reduction*. To evaluate the performance of the proposed SGS, the amount of food waste discharged by the inhabitants of Gangnam district was analyzed. Because the proposed SGS provides a Web-based service, the amount of food waste can be easily analyzed through statistical data collected by the Web-based service.

As mentioned before, 136 SGBs were deployed in the Gangnam district, which consists of six subdistricts. The bins were applied to an apartment housing area in five subdistricts and to detached housing areas in the other district. The adaptive user-oriented charge policy was also applied to the SGS.

**Figure 13:**
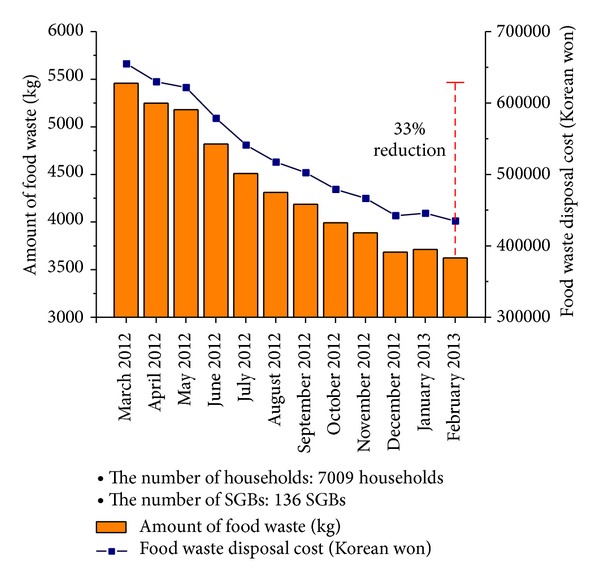
Amount of food waste discharged by the inhabitants of Gangnam district and disposal cost according to the amount of food waste for a one-year period.

Figure 13 shows the amount of food waste discharged by the inhabitants of Gangnam district and disposal cost according to the amount of food waste for a one-year period. There was not a notable result for the initial three months but significant reduction results occurred from June 2012, when an effect of the adaptive user-oriented charge policy appeared. In the last month, the amount of food waste generated per month was decreased by about 33%.

Of course, it is somewhat difficult to consider that the decreased amount accurately shows the performance of the SGS owing to the reliability of a conventional collection system. In addition, the reduction in food waste may be a temporary phenomenon caused from an aversion to the new system. If the reduction occurred constantly, the expectation effectiveness because of the reduction caused by the Pay as You Throw (PAYT) system based on RFID would be expected to be expanded.

## 6. Conclusions and Future Works

In this paper, we proposed an IoT-based SGS for replacing existing RFID-based garbage collection systems. To provide differentiation from passive collection bins and other types of RFID-based food garbage collection systems, we also proposed components required in external and public environments and designed the SGS based on these components. The basic system structure of a SGB is a centralized structure in which information gathered in each bin is transferred to the server; we also designed a HSGB for improving the battery efficiency of each SGB.

An adaptive user-oriented charge policy is used to motivate residents to reduce their food waste, and Web-based services are provided to achieve more efficiency in the disposal and collection processes. Furthermore, based on the proposed system using SGBs, we implemented the proposed system in Gangnam district for a one-year period as a pilot project and verified the results. The energy efficiency of the proposed SGBs shows 16% energy saving result, which shows that SGBs can contribute to not only a reduction of food waste but also energy saving. The proposed system along with the adaptive user-oriented charge policy resulted in a reduction of food waste of about 33%, and it is expected that the proposed system will thereby improve the efficiency of food waste management.

Nevertheless, the proposed SGS requires more maintenance cost than the existing system, and there is a tradeoff owing to the proposed system's battery-based power structure. The most important issue is how to improve the battery life of a SGB. To solve this problem, photovoltaic power generation [15] is being considered. Moreover, high-intensity plastic materials are also being considered for durability against external impact and corrosion from humidity.

## Figures and Tables

**Table 1:** Comparison of the three types of volume-rate garbage disposal systems.

Type	Pros	Cons
Plastic garbage bags	(i) Convenient discharge (ii) High adaptability in poor environments	(i) Inaccurate measurements (ii) Odor problems (iii) Spoils the beauty of the city

Chips and stickers	(i) Remedies for the shortcomings of plastic garbage bags (ii) Various charge commissioning methods	(i) Inaccurate measurements (ii) Elaborate charge commissioning system required (iii) Inconvenient discharge and bin management

RFID-based garbage collection system	(i) Accurate weight measurement (ii) High impact on food waste reduction	(i) Causes server overload owing to data concentration (ii) Low mobility from a fixed power supply (iii) User inconvenience caused by complex discharge process

**Table 2:** Specifications of a smart garbage bin.

	Specification
Type of scale	Substructure
Size	590 × 680 × 1170 mm
Battery capacity	Lithium-ion battery, 7.4 V, 92.4 Ah
Communication	CDMA2000 EV-DO
Type of RFID	ISO 14443, frequency: 13.56 MHz
Weight	Maximum weight: 105 kg, weight unit: 50 g

**Table 3:** Experimental results of Group A.

SGB	Number of uses	Battery remains (%)	Power consumption (Wh)
1	161	29.93	479
2	151	28.99	485
3	197	10.89	609
4	204	8.26	627
5	196	6.59	638
6	144	38.02	423
7	202	6.096	642
8	156	27.51	495
9	177	22.59	529
10	169	23.77	512

**Table 4:** Experimental results of Group B.

SGB	Number of uses	Battery remains (%)	Power consumption (Wh)
1	211	20.07	546
2	199	26.75	500
3	172	35.96	437
4	223	12.50	598
5	192	28.94	485
6	154	39.97	410
7	180	31.35	469
8	207	18.28	558
9	171	37.08	430
10	181	31.47	468
